# Revisiting the SSRI vs. placebo debate in the treatment of social anxiety disorder: the role of expectancy effects, neural responsivity, and monoamine transporters

**DOI:** 10.3389/fpsyg.2025.1531725

**Published:** 2025-05-12

**Authors:** Tomas Furmark, Kurt Wahlstedt, Vanda Faria

**Affiliations:** ^1^Department of Psychology, Uppsala University, Uppsala, Sweden; ^2^Department of Medical Sciences, Uppsala University, Uppsala, Sweden; ^3^Department of Anesthesia, Critical Care and Pain Medicine, Pain and Affective Neuroscience Center, Boston Children's Hospital, Harvard Medical School, Boston, MA, United States; ^4^Interdisciplinary Pain Center, University Hospital and Faculty of Medicine Carl Gustav Carus, TU Dresden, Dresden, Germany

**Keywords:** placebo, selective serotonergic reuptake inhibitors (SSRI), expectancies, social anxiety, neuroimaging, positron-emission tomography

## Abstract

Selective serotonin reuptake inhibitors (SSRIs), widely used for anxiety and depression, are often criticized for their perceived similarity in efficacy to placebo treatments and the unclear connection between brain serotonin levels, on one hand, and the symptomatology of these disorders, on the other. In this perspective paper we discuss the complex mechanisms behind SSRI and placebo treatments in managing social anxiety disorder (SAD), focusing on both pharmacological and expectancy effects. Through a series of neuroimaging studies using positron emission tomography (PET), we investigated the neural, neurochemical and behavioral changes associated with SSRI and placebo responses in SAD patients. Results from one study revealed that both SSRI and placebo responders showed equal reductions in amygdala activity, a region central to fear processing, as well as comparable improvements in social anxiety symptoms. These findings suggest shared neural pathways between SSRIs and placebos, possibly related to response expectancies. In another study, we manipulated patient expectations using a deception design, showing that overt SSRI treatment yielded greater symptom reduction than covert administration. PET results further underscored the influence of expectation on dopamine signaling. Furthermore, PET data on serotonin transporters indicated that serotonin reuptake inhibition alone does not fully account for SSRIs' clinical efficacy, as serotonin transporter occupancy was not correlated with symptom improvement. In yet another study, combining SSRIs with cognitive-behavioral therapy (CBT) led to more robust and longer-lasting outcomes than placebo combined with CBT, with distinct effects on brain monoamine transporters. Overall, these findings emphasize the intricate interplay between pharmacology, brain mechanisms, and psychological expectations in the treatment of SAD.

## Introduction

Selective serotonin reuptake inhibitors (SSRIs) are the most commonly prescribed antidepressants and frequently used to treat anxiety disorders (Lee and Stein, 2023), which are among the most prevalent mental health conditions globally (Yang et al., 2021). Meta-analyses indicate that SSRIs are effective in treating both depression and anxiety (Cipriani et al., 2018; Jakubovski et al., 2019), but debate persists about the magnitude of their therapeutic effects and the extent to which these effects can be attributed to expectancy-driven placebo responses (Kirsch, 2019; Oronowicz-Jaśkowiak and Babel, 2019) and their precise impact on serotonin neurotransmission (Moncrieff et al., 2023; Frick et al., 2015, 2016).

Numerous studies show that patient expectations about treatment effectiveness can significantly impact health outcomes (Mohamed Mohamed et al., 2020; Laferton et al., 2017; Bingel, 2020; Colloca et al., 2004). For example, this has been demonstrated with study designs like the open-hidden paradigm, where participants are either aware or unaware that they are receiving treatment (Colloca et al., 2004). For SSRIs, researchers have argued that a substantial portion of the perceived therapeutic benefit in double-blind randomized clinical trials (RCTs) may stem from placebo responses rather than the drug itself (Sugarman et al., 2014; Fournier et al., 2010; Kirsch, 2014; Moncrieff and Kirsch, 2015). In particular, side effects associated with SSRIs can inadvertently reveal participants' group assignments, undermining the blind. Both patients and clinicians often correctly guess whether they are in the active drug or placebo group, likely enhancing responses in the drug group and reducing responses in the placebo group (Chen et al., 2011, 2015; Hróbjartsson et al., 2014; Margraf et al., 1991; Baethge et al., 2013). However, other scholars argue that SSRIs' perceived limitations may stem from the use of compromised outcome measures (Hieronymus et al., 2016), or neglecting lack of association between adverse event severity and clinical response (Hieronymus et al., 2018).

Another point of contention is the “serotonin deficit hypothesis” which postulates lowered serotonin in affective disorders and that SSRIs alleviate symptoms by normalizing serotonin levels (Moncrieff et al., 2023). While SSRIs block serotonin uptake by inhibiting the serotonin transporter protein (Meyer et al., 2004), this action doesn't consistently correlate with symptom improvement (Cavanagh et al., 2006; Hjorth et al., 2021). Moreover, although serotonin transporter occupancy occurs within hours of SSRI administration, clinical effects are delayed by weeks (Baldinger et al., 2014), suggesting that other mechanisms are involved. It also remains unclear whether SSRI-responsive disorders like depression and anxiety stem from serotonin underactivity or overactivity (Frick et al., 2015; Andrews et al., 2015). Downstream effects—such as those on dopamine neurotransmission, implicated in approach-avoidance motivation—may also play a key role. Interactions between dopamine and serotonin signaling may be important for both anhedonia and heightened sensitivity to, and avoidance of, threatening stimuli (Boureau and Dayan, 2011).

Understanding SSRI efficacy, the proportion of the effect due to pharmacological vs. expectancy factors, and the neural mechanisms underlying symptom remission are essential areas for future research. Central to this debate is the influence of expectancy and beliefs—key components of the placebo effect—on treatment outcomes. In our research we have used neuroimaging techniques to explore these questions in social anxiety disorder (SAD), a condition marked by excessive fear of scrutiny in social performance or interaction situations. SAD is one of the most common anxiety disorders and impose a considerable burden on patients and society (Frick et al., 2015). While SSRIs are effective for treating SAD, the placebo response can be substantial (Baldwin et al., 2016). Our goal has been to investigate SSRI efficacy and underlying brain mechanisms in comparison to placebo and other non-pharmacological interventions like cognitive-behavioral therapy (CBT).

In this paper, we will discuss four major findings from our neuroimaging treatment studies and their implications for the broader SSRI vs. placebo debate. All studies are in the public domain and there are no secondary analyses.

## Responders to SSRIs and placebo show equal improvement and shared neural response phenotypes

Effective prevention and treatment of anxiety disorders would benefit from a deeper understanding of the neurobiological factors involved, and it's unclear whether SSRIs and placebos engage similar or different neural mechanisms. In the first neuroimaging study discussed here, we investigated amygdala activity and connectivity changes linked to symptom improvement in SAD patients treated with SSRIs or pill placebo (Faria et al., 2012, 2014). The amygdala is a crucial target for anxiolytic treatments due to its central role in fear processing, its connections with cortical regions involved in emotion regulation, and its heightened reactivity to emotional stimuli in patients with anxiety disorders (Etkin and Wager, 2007). However, the amygdala is often mistakenly seen as a single unit, complicating the identification of treatment-specific brain changes. Additionally, successful SSRI and placebo treatment may involve different timing and amygdala-frontal connectivity patterns.

### Study outline and results

Neuroimaging data were extracted from three randomized, double-blind, placebo-controlled trials, including SSRI and placebo treatment outcome data from 72 SAD patients. Regional cerebral blood flow (rCBF), indexing synaptic activity, was measured with positron emission tomography (PET) during an anxiety-provoking public speaking task, with both brain scans and public speaking task repeated after 6–8 weeks of treatment (Faria et al., 2012). Clinical response was determined by the Clinical Global Impression improvement item (CGI-I) (Zaider et al., 2003) and the Liebowitz Social Anxiety Scale (LSAS) (Liebowitz, 1987).

**Figure 1:**
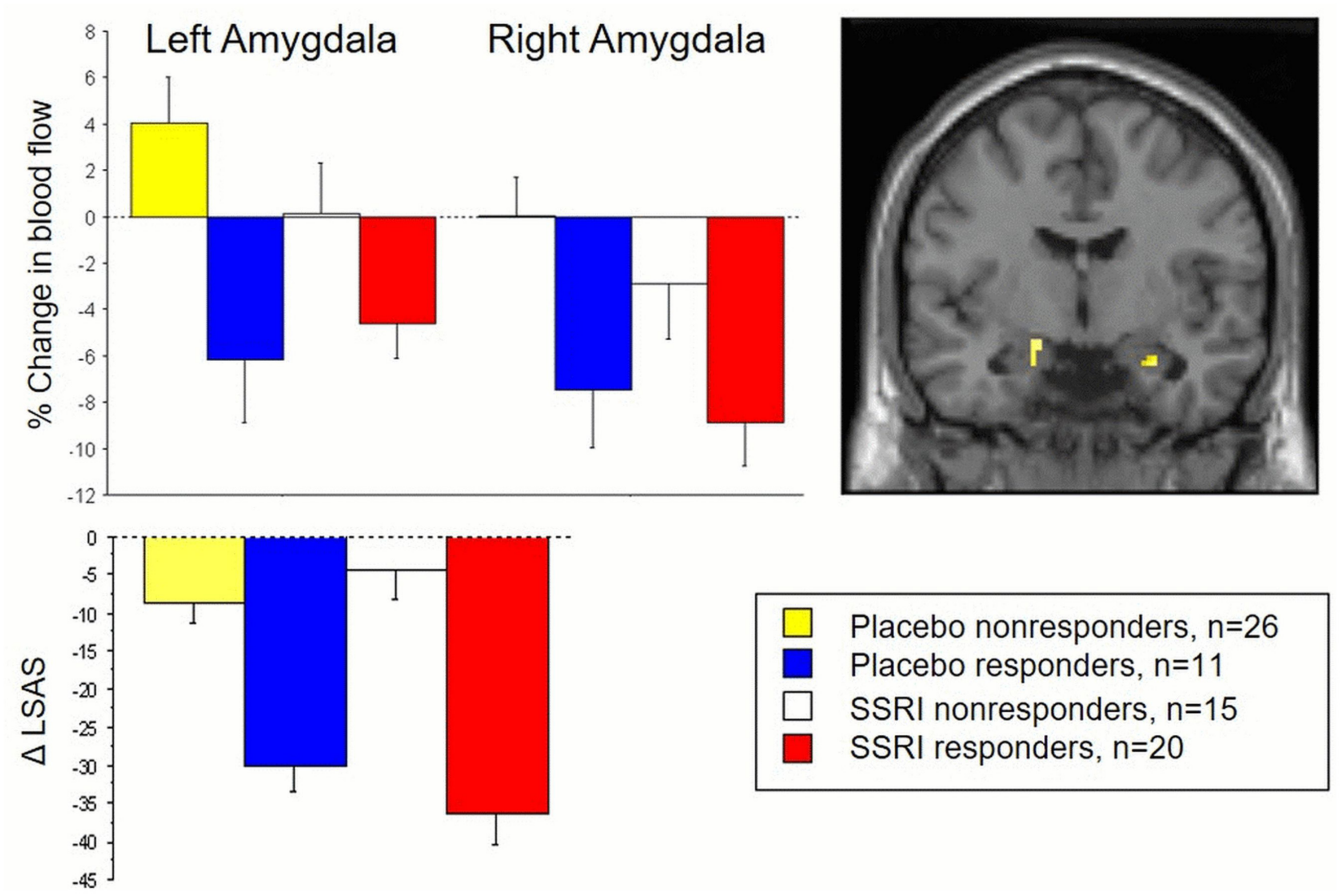
The **top panel** displays changes in amygdala activity, measured by PET and regional cerebral blood flow, during an anxiety-provoking public speaking task in both responders and non-responders to SSRI and placebo treatment for social anxiety. The **lower panel** presents corresponding behavioral outcomes, with reduced social anxiety measured by the Liebowitz Social Anxiety Scale (LSAS) as a function of treatment. Both SSRI and placebo responders exhibited common reductions in amygdala activity and social anxiety, clearly distinguishing them from the non-responder subgroups at both the brain and behavioral levels. See Faria et al. (2012) for details.

Results showed common attenuation of anxiety-related neural activity in SSRI as well as placebo responders in subregions of the amygdala, corresponding to the left basolateral and right ventrolateral parts (Faria et al., 2012). The neural changes in these parts of the amygdala correlated with behavioral measures of reduced anxiety after treatment and differentiated responders from non-responders. Importantly, no significant differences in amygdala attenuation were found between SSRI and placebo responders, and clinical improvement was comparable (see Figure 1).

Follow-up analyses of altered amygdala connectivity patterns also suggested shared anxiolysis-related connectivities between the amygdala and several frontal-cortical regions in SSRI and placebo responders (Faria et al., 2014). There was shared inverse co-activation, i.e., negative connectivity, between the left amygdala, on one hand, and dorsolateral prefrontal cortex and rostral anterior cingulate cortex, on the other hand. There was shared positive connectivity between the left amygdala and dorsal anterior cingulate cortex.

### Discussion

These results indicate that SAD patients who responded well to placebo showed equally large clinical improvement, similar attenuation of stress-related amygdala reactivity, and comparable alterations in amygdala-frontal connectivity as SSRI responders. Thus, SSRI and placebo responders share overlapping neuromodulatory paths that may underlie improved emotion regulation and reduced anxiety. These results align with other brain imaging trials on SAD suggesting that amygdala attenuation differentiates placebo responders from non-responders (Furmark et al., 2008) and also represents a common neural pathway both for SSRIs and CBT (Furmark et al., 2002). Interestingly, such attenuation has been linked to serotonin-related gene variants (Furmark et al., 2008). Shared neural profiles in SSRI and placebo responders have been found also in depression (Mayberg et al., 2002).

At first glance, similar improvement and shared neural response phenotypes in SSRI and placebo responders may fuel criticism against SSRIs, claiming these drugs have indistinguishable effects compared to placebos. However, the number of SSRI responders was higher than for placebo (57 and 30% respectively) but, since an active placebo was not used, this could be related to the “breaking of the blind effect” (Kirsch, 2014). Nonetheless, SSRI and placebo responders may still differ in brain functions or neurotransmission systems that were not measured.

## Expectancies shape the outcome of SSRI pharmacotherapy through altered dopamine signaling

In a subsequent neuroimaging project, our goal was to further investigate treatment mechanisms underlying SSRI vs. placebo responses by examining their relationship with brain neurotransmitter functions, focusing on serotonin and dopamine transporter proteins assessed with PET imaging. We used a research design involving deception to manipulate expectations in SAD patients during SSRI treatment. This allowed us to disentangle the placebo effect from the pharmacological impact of the treatment (Hjorth et al., 2021; Faria et al., 2017).

Expectations are crucial in treatment evaluation and pose challenges for drug development. Double-blind RCTs fail to fully account for expectations when patients and doctors can guess treatment based on side effects. The balanced placebo design (Ross et al., 1962) offers a potential solution by incorporating four conditions: told drug–receive drug, told placebo–receive drug, told drug–receive placebo, and told placebo–receive placebo. Due to ethical and practical considerations, our study employed only the first two conditions. The objective was to explore how the effect of escitalopram varies when overtly administered with accurate information compared to covert administration, with the incorrect information that the drug is an “active placebo.”

The comparison between overt and covert SSRI treatment groups allows for estimating the placebo/expectancy effect, without actually administering a placebo, and PET assessments provide deeper insights into neurotransmitter-level changes. Thus, patients with SAD underwent PET scans before and after treatment, using radioligands designed to target serotonin and dopamine transporters.

### Study outline and results

A total of 46 SAD patients participated (Faria et al., 2017), with a subset of 27 participants undergoing PET scans (Hjorth et al., 2021). Fourteen PET participants received escitalopram overtly, while 13 received the drug covertly in capsule form along with a cover story. Thus, the experimental manipulation involved two SSRI groups. The overt group received accurate information from the psychiatrist, stating they would take escitalopram (10 mg/day for the first week, then 20 mg/day for 8 weeks). The covert group was falsely told they would receive an “active placebo” (a neurokinin-1 antagonist) mimicking escitalopram's side effects. Importantly all patients received the same dosage of 20 mg of escitalopram. All participants were instructed to keep their treatment confidential. The psychiatrist contacted participants after 1 week and again at the study's conclusion. Symptom changes were assessed online using the self-report version of the LSAS (Liebowitz, 1987). To address ethical concerns related to deception, all participants were offered CBT after the initial treatment period.

**Figure 2:**
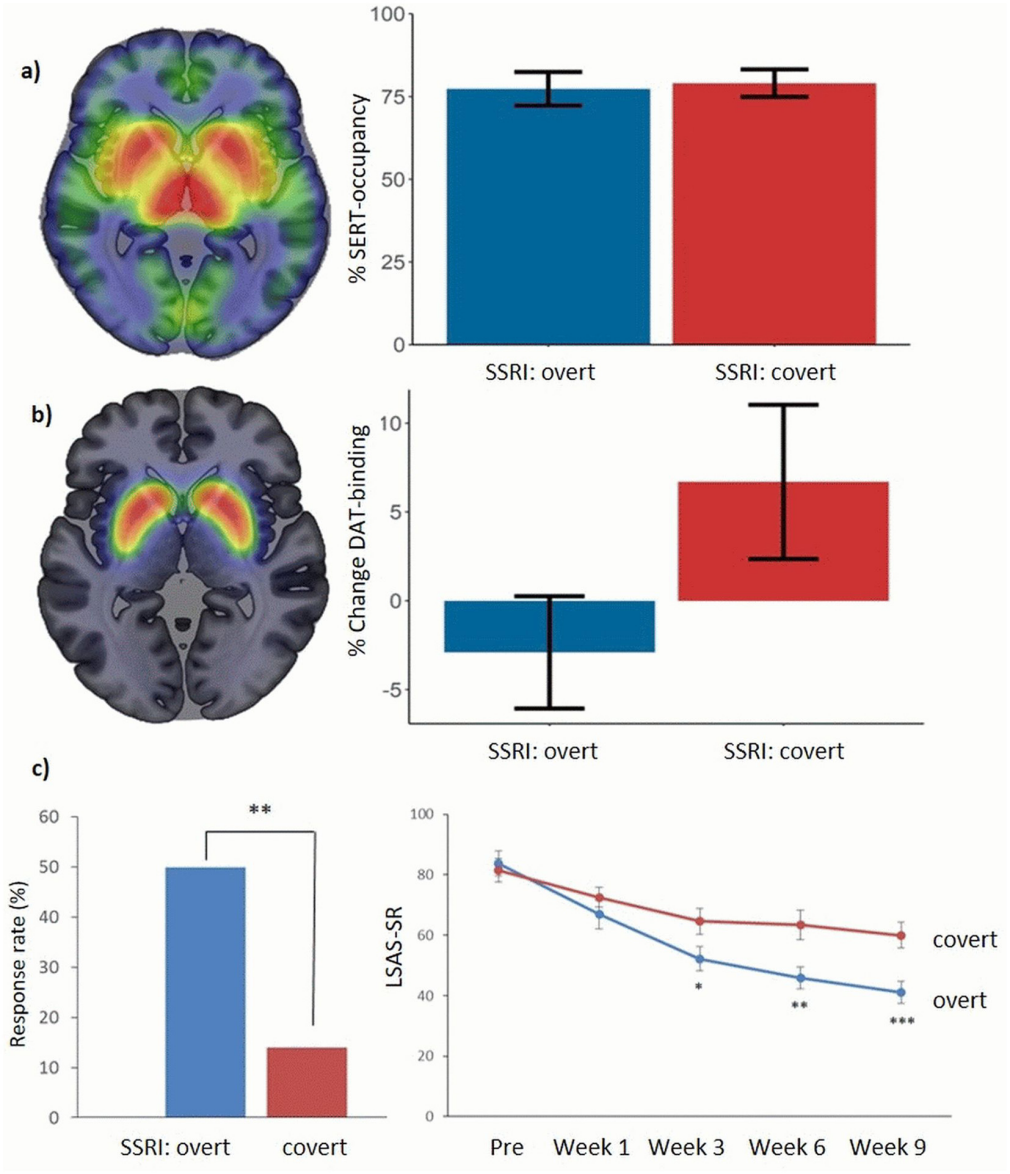
**(a)** PET scans of serotonin transporters in patients with social anxiety disorder, using the tracer [11C]-DASB, before treatment (left) and treatment effects (right). After treatment, an equally high average occupancy of serotonin transporters (altered binding) was observed with overt and covert SSRI treatment. No group differences were present before treatment. See Hjorth et al. (2021) for details. **(b)** Corresponding PET scans of dopamine transporters, using the tracer [11C]-PE2I, before treatment (left) and treatment effects in the putamen/pallidum (right). After treatment, a decrease in dopamine transporter availability was observed in the overt SSRI group, while increases were seen in the covert SSRI group. No group differences were present before treatment. See Hjorth et al. (2021) for details. **(c)** Percentage of responders and symptom improvement during treatment in the two groups as measured with the Liebowitz Social anxiety scale self-report (LSAS-SR) in the whole sample. See Faria et al. (2017) for details; *p < 0.05, **p < 0.01, ***p < 0.005.

After SSRI-treatment, both groups showed symptom improvements, but the overt SSRI group had significantly greater improvement with twice the effect size (Cohens d = 2.24 vs. 1.13). This difference was evident by week 3 and increased over time. Also, a higher percentage of patients in the overt group showed clinically significant improvement (50 vs. 14%, p <0.01). The within-group effect of covert SSRI administration was nonetheless superior to that of a previously assessed waiting-list control group (*p* < 0.001) (Faria et al., 2017). In the subsample (Hjorth et al., 2021), PET scans revealed differences in dopamine transporter availability, correlating strongly with symptom improvement (*p* < 0.001, R = −0.61): decreases were noted in the putamen and pallidum for the overt SSRI group whereas increases were observed in the covert group – see Figure 2.

### Discussion

The SSRI treatment effect on social anxiety was strongly linked to expectations, a key aspect of the placebo effect. With lower expectations, the clinical effect was substantially diminished, and the placebo effect accounted for about half of the symptom improvement. These findings align with previous studies on the role of expectations (Bingel, 2020; Bingel et al., 2011), highlighting the influence of doctor-patient interactions. The PET results suggest that dopamine plays a significant role in the expectancy effects observed in SSRI treatment outcomes. Overt SSRI treatment was linked to decreased dopamine transporter availability, correlating with symptom improvement. This decrease may reflect increased dopamine release, leading to more bound transporters. Since escitalopram alone cannot account for this effect on dopamine transporters, it likely stems from differing expectations about the treatment's effectiveness. These findings also align with previous studies suggesting a critical role of dopamine in placebo responsivity (Scott et al., 2008). Ideally, future studies should include all four cells of the balanced placebo design (Ross et al., 1962) including the told drug-receive placebo condition.

## Serotonin reuptake inhibition alone is insufficient for the clinical efficacy of SSRIs

Our PET data on serotonin transporters, in the above-mentioned study, allowed us to explore the relationship between serotonin reuptake inhibition and clinical outcomes following escitalopram treatment (Hjorth et al., 2021). SSRIs are believed to exert their effects by inhibiting the serotonin transporter, with a typical occupancy rate of approximately 76–85% required for robust clinical effects (Meyer et al., 2004). However, a key question is whether serotonin transporter occupancy after prolonged SSRI treatment correlates with treatment response—does it differ between SSRI responders and non-responders? This question is also relevant to the serotonin deficit hypothesis of affective disorders, which has been central to the SSRI vs. placebo debate (Moncrieff et al., 2023).

### Study outline and results

We analyzed serotonin occupancy rates based on PET measures in the deception study comparing overt vs. covert SSRI treatment as previously described (Hjorth et al., 2021). There was no difference in serotonin transporter binding between the groups at the start of the study or the degree of binding at the end of the study (average serotonin transporter occupancy 78%); see Figure 2. Also, there was no correlation between serotonin transporter occupancy, i.e., the degree of reuptake inhibition, and social anxiety improvement. Additionally, there were no significant correlations between symptom improvement and the concentration of escitalopram or its metabolites in the blood.

### Discussion

These results suggest, first, that both groups adhered to the prescribed SSRI regimen, as confirmed also by blood analyses of drug concentrations and metabolites. Second, they indicate that the clinical effect of SSRIs is not solely mediated by serotonin reuptake inhibition. Since both the overt and covert SSRI groups had similar serotonin transporter occupancy rates but significantly different treatment outcomes, SSRIs cannot exert their anxiolytic effects through reuptake inhibition alone.

These findings add to the ongoing debate challenging the serotonin deficit hypothesis (Moncrieff et al., 2023), which, however, has long been considered overly simplistic (Owens, 2004). Our results do not rule out other serotonergic mechanisms with SSRIs, such as altered synthesis rate (Frick et al., 2016) or the possibility that effective treatment of social anxiety may influence serotonin signaling indirectly, through pathways beyond transporter blockade. Indeed, SSRIs can induce a variety of neuroplastic effects as well as changes in cognitive and emotional processing (Page et al., 2024). The dopamine-related expectancy effects discussed earlier also suggest that serotonin-dopamine interactions may play a role in SSRI therapeutic mechanisms. A better understanding of all these possible mechanisms could be gained by comparing SSRIs with effective non-pharmacological treatments like CBT at the neurotransmitter level, which we now turn to.

## Pharmacological and psychological treatments have distinct effects on brain monoamine transporters

In contrast to the previous project, which focused on separating expectancy effects from SSRI drug effects, we conducted another PET study aimed at identifying the added value of SSRIs when combined with psychological treatment. Our goal was to examine changes in brain monoamine transporter binding following combined SSRI and CBT treatment, compared to placebo combined with CBT (Hjorth et al., 2022). CBT is the first-choice psychotherapy for SAD and is generally considered as effective as SSRIs (Mayo-Wilson et al., 2014). This study allowed us to investigate the specific neurochemical changes associated with adding SSRIs to psychological therapy and thus to explore differences between SSRI-exposed and unexposed patients.

### Study outline and results

This was a double-blind RCT in which half the participants received clinical doses of escitalopram for 9 weeks, while the other half received placebo, both administered covertly in capsules (Gingnell et al., 2016). Both groups underwent an internet-based CBT program (Andersson et al., 2006) during the 9-week period. The CBT was supported by experienced therapists, who provided weekly assignments and feedback via email. A subset of SAD patients underwent PET scans before and after treatment (Hjorth et al., 2022). A total of 24 individuals completed the full treatment and neuroimaging assessments, using the same PET radioligands for serotonin and dopamine transporters as previously described, i.e., two scans at each time point.

Treatment results showed that both the escitalopram + CBT and placebo + CBT combinations yielded significant improvement (*p* < 0.001), with social anxiety symptom assessments on the LSAS-SR decreasing by 37 and 24 points, respectively (Hjorth et al., 2022). Furthermore, as reported in the main study involving the full treatment sample (48 patients) (Gingnell et al., 2016), better long-term effects measured after 15 months were observed in the escitalopram + CBT group (*p* = 0.03, Cohen's *d* = 1.58 vs. 0.87). Initial treatment credibility ratings and expectancy of improvement did not differ across groups, and here participants could not guess their allocated treatment better than chance.

The PET results showed that over 80% of serotonin transporters were occupied in the escitalopram + CBT group, indicating adherence to the medication. No serotonin reuptake inhibition was seen after placebo + CBT; on the contrary, serotonin transporter availability increased in the raphe nuclei. In the SSRI + CBT group, reduced anxiety correlated with increased striatal serotonin transporter binding, but not with blood drug concentration. Decreased dopamine transporter availability in the nucleus accumbens and left thalamus correlated with reduced social anxiety, whereas an opposite correlation was observed in the placebo + CBT group (Hjorth et al., 2022).

### Discussion

While both treatment combinations led to significant improvements, the SSRI + CBT group showed better outcomes, with more responders and superior long-term results. Given the effective blinding and equal initial credibility ratings, it is unlikely that patient expectations influenced these differences. Blockade of serotonin transporters was observed only in SSRI-exposed individuals. As in the deception study (Hjorth et al., 2021), decreased dopamine transporter availability correlated with symptom improvement in this group. However, this association may not apply to all treatment modalities, as the placebo + CBT group showed the opposite correlation. The reason remains unclear, but it should be noted that the study was not designed to isolate CBT's specific effects, and expectations were not manipulated. CBT likely produces complex psychological effects involving emotion, cognition, and motivation. Nonetheless, the findings suggest that improvement from pharmacological (SSRI-exposed) vs. psychological (SSRI-unexposed) treatments relies on distinct monoaminergic mechanisms. This difference at the neurotransmitter level was in contrast to our imaging activation studies showing common changes in amygdala responsivity across treatment modalities (Faria et al., 2012, 2014; Furmark et al., 2008, 2002).

## General discussion and conclusions

### What are the key mechanisms underlying successful treatment outcomes?

While serotonin reuptake inhibition is a specific effect of SSRIs, it has weak ties to clinical improvement. In contrast, our data suggest that expectancy-related effects, impacting dopamine transporters, are significantly associated with symptom improvement and account for much of the variance in SSRI treatment outcomes. As demonstrated by Price and Anderson (2012), expectations likely contribute to CBT outcomes as well; however, we did not manipulate expectations related to CBT in our study.

Determining precise treatment mechanisms, however, remains very complex. The studies discussed here provide insights but only contribute a few pieces to the overall puzzle. Further research is needed to elucidate how altered dopamine signaling contributes to treatment outcomes, how different treatments modulate serotonin–dopamine interactions and the time course of changes. This includes research of the amygdala–striatal circuitry and the broader neural networks instantiating approach–avoidance motivation. Much work is needed to ensure that today's evidence-based treatments are not only effective, but also firmly grounded in robust data and well-supported theories of etiology, pathology, and healing processes.

### What are the implications for the SSRI vs. placebo debate?

Firstly, while the findings discussed here may raise concerns from a pharmaceutical perspective, there is no reason to be overly critical of SSRIs or to dispute their status as evidence-based treatments for anxiety disorders. Even under challenging conditions, such as when administered with incorrect information, the within-group effect size of covert SSRI treatment was substantial, and superior to untreated waiting-list controls (Faria et al., 2017). Thus, we do not claim that SSRIs are lacking anxiety-reducing properties. However, the drug itself cannot take full credit for the improvement observed in RCTs, and explanations beyond serotonin reuptake inhibition are clearly needed to fully understand their therapeutic benefits.

Secondly, expectation effects, shaped by the information patients receive, are measurable in the brain and play a significant role in treatment outcomes. The expectation of improvement can be further enhanced through factors such as attention, engagement, feeling well-treated, and participating in therapeutic rituals (Benedetti and Amanzio, 2011). These elements provide comfort, instill hope, and improve motivation and adherence to treatment. How the treatment is presented by the doctor or therapist can be as important as the treatment itself. This also includes addressing side effects and managing negative expectations, i.e., nocebo effects.

## Data Availability

The original contributions presented in the study are included in the article/supplementary material, further inquiries can be directed to the corresponding author.
